# Lower serum FT3 within the reference range is associated with mortality for older adults over 80 years of age with sarcopenia

**DOI:** 10.1186/s12877-023-03783-8

**Published:** 2023-02-06

**Authors:** Li Zhang, You-Yi Tu, Zhe Zhao, Jun Jin, Jun Tao, Xiao-Yan Zhang

**Affiliations:** Department of Geriatrics, Shanghai Sixth People’s Hospital Affiliated to Shanghai Jiao Tong University School of Medicine, No. 600, Yi Shan road, Shanghai, 200233 China

**Keywords:** Aging, FT3, Sarcopenia, Mortality

## Abstract

**Objective:**

Thyroid hormones stimulate myogenesis and muscle contraction and regulate skeletal muscle cell metabolism. However, the association between thyroid hormone levels and mortality in sarcopenic older adults remains elusive. The aim of this study was to investigate the relationship between thyroid hormones and all-cause mortality in people over 80 years of age with sarcopenia.

**Methods:**

This study was performed on 264 sarcopenic patients aged 80 years and older. Serum levels of thyroid hormone, including free triiodothyronine (FT3), free thyroxine (FT4), and thyroid stimulating hormone (TSH) were tested to evaluate thyroid status. Sarcopenia was defined using the criteria of the European Working Group on Sarcopenia in Older People. Mortality data were available for up to 38 months of follow-up. The correlation between FT3 and calf circumference (CC) or handgrip strength (HGS) was determined by Pearson correlation analysis. Kaplan-Meier analysis was used to compare the differences between FT3 tertile groups. Cox regression was used to analyze the mortality risk ratio of patients with different FT3 tertiles.

**Results:**

During the follow-up period, 88 older adults died. Non-Survivors had lower serum FT3 levels (3.7 ± 0.5 vs. 3.9 ± 0.7, *P* = 0.001) than the Survivor. Serum FT3 was positively associated with CC and HGS (*r* = 0.29, *P* < 0.001, *r* = 0.21, *P* = 0.002, respectively). The Kaplan-Meier curve analysis demonstrated a difference in mortality among the FT3 tertile groups (log-rank test, χ^2^ = 11.83, *P* = 0.003). The high FT3 group had lower mortality compared with the low FT3 group (the adjusted HRs were 0.63 (95%CI: 0.41–0.96 *P* = 0.031).

**Conclusion:**

Lower FT3 within the reference range is associated with higher mortality in adults over 80 years with sarcopenia and euthyroid. Routine assessment of FT3 may be an easy way to identify high-risk older adults with sarcopenia.

**Supplementary Information:**

The online version contains supplementary material available at 10.1186/s12877-023-03783-8.

## Introduction

Sarcopenia, an age-related reduction of muscle mass, strength, and function, has become a global health concern due to its influence on loss of independence, health care cost, and other adverse health outcomes [1]. Although sarcopenia has been recognized as an independent condition with its code (M62.84) in ICD-10CM [2], it is still not fully recognized and managed in routine clinical practice [3]. Since sarcopenia is usually associated with an increased risk of death, identifying biomarkers that predict mortality in patients with sarcopenia is essential to improve clinical outcomes.

Endocrine changes with aging involve almost all glands. Changes in hormone levels with aging lead to mass muscle reduction and decreased strength, including testosterone, growth hormone, thyroid hormone, vitamin D, and insulin-like growth factor [4]. Thyroid hormones exert an essential control on the mitochondria function and induce the transition from a slower fiber type into a faster one [5], while mitochondria dysfunction and faster fibers reduction are the main mechanisms of sarcopenia [6]. Studies have shown that dominant thyroid disease is associated with decreased muscle mass and strength. Brennan MD and colleagues reported that before treatment, compared with patients with normal thyroid function, adults with obvious hyperthyroidism had lower muscle strength and smaller midthigh sectional area. In addition, six to nine months after restoring normal thyroid status, the muscle parameters of both changes have improved [7].

Free Triiodothyronine (FT3) is the active form of the thyroid hormones, which can stimulate muscle formation and contraction and regulate skeletal muscle cell metabolism [8]. A significant decrease in FT3 is expected in the older adults with acute disease or undergoing surgery. However, a slight decrease in FT3 levels can also be found in healthy older people [9]. Recently, a study including 6974 participants from Brazil reported a negative relationship between FT3 with muscle mass in middle-aged and older adults with euthyroid, subclinical hypothyroidism, and subclinical hyperthyroidism [10]. Another study from China reported that relatively higher FT3 levels in the reference were associated with muscle mass and muscle function in older adults [11].

It has been proved that sarcopenia is a predictor of all-cause mortality in the older adults [12]. Our previous study found that the NRS2002 score is an independent predictor for all-cause mortality in the sarcopenia geriatrics population [13]. The National Health and Nutrition Examination Survey found that a lower FT3 level was an independent predictor of cardiovascular mortality in 7116 adults with a median 45-month follow-up [14]. In a prospective cohort study, FT4 and FT3 levels in the normal range were negatively correlated with cancer mortality and all-cause mortality [15]. There might be a potential relationship between thyroid hormones and the prognosis of sarcopenia. A thyroid hormone test is quite regular for older adults in many countries, even included in physical examination. If the routine evaluation of FT3 is an easy method to identify high-risk patients with sarcopenia, early intensive intervention may be helpful to improve the outcome of these patients.

As far as we know, there were no longitudinal studies focused on the performance of thyroid hormone on mortality in sarcopenia. Sarcopenia becomes increasingly common with age, yet few studies focus on older adults over 80 years, although it has been reported that sarcopenia affects up to 50% of people over 80 years [16]. The aim of this study was to assess the relationship between thyroid hormone levels within the normal range with mortality among geriatric euthyroid subjects with sarcopenia during the follow-up periods.

## Methods

### Study population and design

A total of 1026 consecutive ≥80 years older adults were screened from the geriatric department of Shanghai Sixth People’s Hospital Affiliated to Shanghai Jiao Tong University School of Medicine from July 2017 to March 2018. The inclusion criteria were ≥ 80 years old patients with sarcopenia. The exclusion criteria were non-sarcopenia(*n* = 454), history of thyroid disease or routine use of anti-thyroid drugs or thyroid hormones (*n* = 22); other diseases that may have effect on muscle metabolism (e.g., inflammatory myopathy, rheumatoid arthritis, Parkinson’s disease, significant liver or kidney dysfunction)(*n* = 39), and TSH, FT4, or FT3 levels were not within the reference range(*n* = 53), the presence of carcinomatous cachexia (*n* = 37), critical illness (including severe infection, acute stroke, acute myocardial infarction) (*n* = 97), edema (*n* = 12), inability to communicate(*n* = 13), and bedridden status (*n* = 35) (Supplemental Fig. 1). In the end, 264 older adults over 80 years of age with sarcopenia were included in this study. The study was conducted in accordance with the Declaration of Helsinki and approved by the institutional review board of Shanghai Sixth People’s Hospital Affiliated to Shanghai Jiao Tong University School of Medicine.

### Data collection

All the participants’ demographic data, lifestyle information, and chronic disease history were collected through medical records. Chronic diseases included hypertension, diabetes, cerebral infarction, chronic obstructive pulmonary disease (COPD), coronary heart disease (CHD), and neoplasms. The diagnosis criteria of these diseases were referred to in our previous publication [13].

### Anthropometric measurement

Height, body weight, and calf circumference (CC) were collected as anthropometric data. Body Mass Index (BMI) was calculated as body weight in kilograms divided by height in meters squared. CC was measured at the greatest circumference of the lower right leg with the participants standing position. All measurements were performed three times, and the means of the CC were recorded as the final data for analysis.

### Handgrip strength (HGS)

WCS-100 electronic vibrometer was used to test the handgrip strength. The handle was adjusted if necessary. The maximum handgrip strength of the dominant hand was measured three times. Between each test, the participants must take at least 1 minute of rest. The maximum handgrip strength value of three times in kilograms was recorded as the final data.

### Sarcopenia diagnosis

The revised definition of Sarcopenia from European Working Group on Sarcopenia in Older People (EWGSOP2) was used in this study [17]. According to the recommended cutoffs, HGS < 27 kg in men or < 16 kg in women was recognized as low muscle strength. Low muscle quantity was recognized as CC lower than 31 cm. In this study, the participants who had low muscle strength together with low muscle quantity were diagnosed with sarcopenia. All the participants did not accept muscle function evaluation in this study.

### Laboratory measurements

All participants had overnight fasting before the blood samples were taken. Serum FT3, FT4, and TSH were measured by electrochemiluminescence immunoassay (Roche Cobas 6000; Roche Ltd., Basel, Switzerland). Reference ranges were defined as TSH level of 0.27–4.20 mIU/L; FT4 level of 12.0–22.0 pmol/L; and FT3 level of 3.1–6.8 pmol/L. All of them were based on our hospital’s laboratory references. The intra- and inter- assay coefficients of the two indexes were < 10%.

### Follow-up for mortality

All participants were followed up in the geriatrics’ clinic of the Shanghai Sixth People’s Hospital Affiliated to Shanghai Jiao Tong University School of Medicine. The deadline for follow-up action was September 30, 2020. Record all mortality that occurred between the start and end dates of the study. As the subjects received medical care in our hospital, no follow-up was omitted.

### Statistical analyses

For continuous variables, the data was expressed as mean ± standard deviation or median (25–75 percentile). The differences between groups were assessed by student’s t test or Mann Whitney U test. Categorical variables were expressed as percentage of frequency, and chi square test was used for comparison between two groups. The correlation between FT3 and CC or HGS was determined by Pearson correlation analysis. Patients were divided into tertile group according to FT3 levels. Kaplan-Meier analysis combined with log rank test was used to compare the differences between FT3 tertile groups. Cox regression was used to analyze the mortality risk ratio (HR) of patients with different FT3. All statistical analyses were performed using SPSS 21.0 software (SPSS Inc., Chicago, IL). Bilateral *P* value < 0.05 was statistically significant.

## Results

### General characteristics of the participants

Two hundred sixty-four patients (mean age, 85 ± 6 years; male/female, 195/69) were enrolled in this study. Eighty-eight participants passed away during a median 27 months follow-up. The clinical characteristics of the survival sarcopenic patients and non-survivor were presented in Table 1. There was a significant difference in age, sex, CC, the prevalence of cerebral infarction, and COPD prevalence in the two groups (*P* < 0.05). Serum FT3 values were significantly lower in non-survival sarcopenic patients compared to survival sarcopenic patients (3.7 ± 0.5 vs. 3.9 ± 0.7, *P* < 0.001). There was no difference in smoking, drinking, the prevalence of CHD, the prevalence of diabetes, the prevalence of dyslipidemia, the prevalence of hypertension, the prevalence of neoplasm, BMI, HGS, FT4, and TSH (*P* > 0.05).

**Table 1:** Baseline characteristics of survivor and non-survivor with sarcopenia

Variables	Survivor	Non-Survivor	*P*
	*n* = 176	*n* = 88	
Age (years) ^a^	85.9 ± 5.5	87.4 ± 5.2	0.001
Sex (male%)^b^	68.2	85.2	0.003
Smoking (%)^b^	18.4	27.6	0.109
Drinking (%)^b^	2.8	3.4	0.800
CI (%)^b^	40.8	54.5	0.037
COPD (%)^b^	25.3	40.9	0.011
CHD (%)^b^	64.4	69.3	0.490
Diabetes (%)^b^	32.8	33.0	0.989
Hypertension (%)^b^	85.6	86.4	0.872
Dyslipidemia (%)^b^	24.7	21.6	0.646
Neoplasms (%)^b^	17.2	26.4	0.102
BMI (kg/m^2^) ^a^	24.7 ± 4.7	21.8 ± 3.3	0.435
CC (cm) ^a^	29.8 ± 2.4	27.9 ± 3.4	< 0.001
HGS (kg) ^a^	16.2 ± 6.4	15.0 ± 6.03	0.149
FT3(pmol/L) ^a^	3.9 ± 0.7	3.7 ± 0.5	0.001
FT4(pmol/L) ^a^	16.2 ± 2.9	15.7 ± 3.0	0.263
TSH (mIU/L) ^c^	2.1(1.4–3.9)	2.2(1.5–3.7)	0.590

*Abbreviations***:** *CI* Cerebral infarction, *COPD* Chronic obstructive pulmonary disease, *CHD* Coronary heart disease, *BMI* Body mass index, *CC* Calf circumference; HGS: Handgrip strength, *FT*3 Free Triiodothyronine, *FT4* Free Thyroxine, *TSH* Thyroid stimulating hormone

^a^t-test. ^b^ chi-square test. ^c^Mann-Whitney test

### FT3 was positively associated with CC and HGS

As shown in Table 2, FT3 was positively associated with BMI (*r* = 0.26, *P* < 0.001), CC (*r* = 0.29, *P* < 0.0001), and HGS (*r* = 0.21, *P* = 0.002). However, neither FT4 nor TSH was associated with these parameters of sarcopenia in euthyroid old subjects (*P* > 0.05). After controlling for age, sex and BMI, partial correlations analysis showed FT3 level was still positively associated with CC (*r* = 0.24, *P* = 0.002), and HGS (*r* = 0.18, *P* = 0.025).

**Table 2:** Pearson correlation analysis of with anthropometric parameters with thyroid hormones

	FT3		FT4		TSH	
	r	***p***	r	***p***	r	***p***
**BMI**	**0.260**	**< 0.001**	**0.078**	**0.251**	**−0.026**	**0.718**
**CC**	**0.296**	**< 0.001**	**−0.053**	**0.437**	**−0.010**	**0.889**
**HGS**	**0.185**	**0.006**	**0.056**	**0.408**	**−0.118**	**0.096**

*Abbreviations*: *BMI* Body mass index, *CC* Calf circumference; HGS: Handgrip strength, *FT3* Free Triiodothyronine, *FT4* Free Thyroxine, *TSH* Thyroid stimulating hormone

### The relationship between FT3 and mortality

**Fig. 1:**
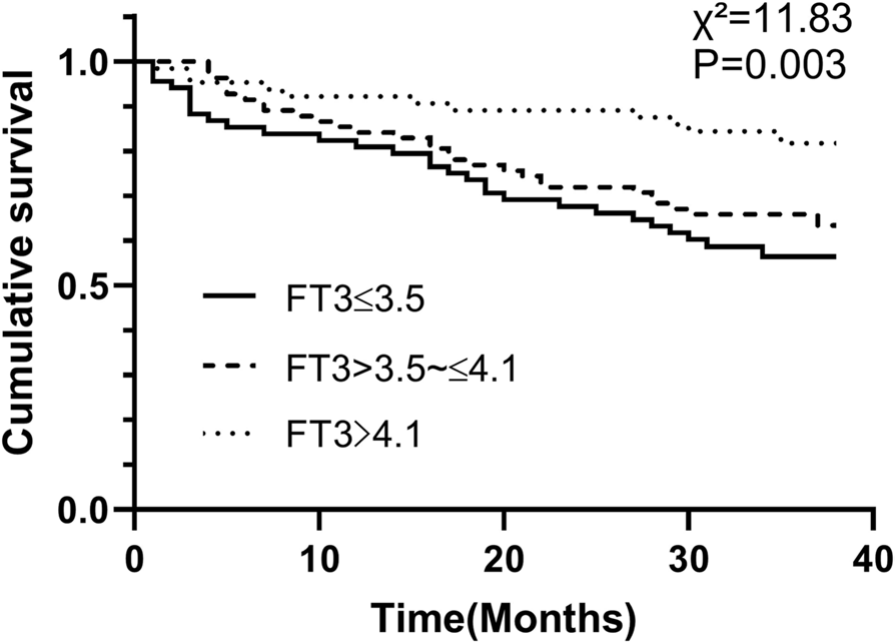
Kaplan-Meier analysis for mortality. Log-rank test χ^2^ = 11.83, *P* = 0.003

The all-cause mortality in serum FT3 levels tertile was compared. The Kaplan–Meier curve demonstrated a significant difference between the three groups (log-rank test, χ^2^ = 11.83, *P* = 0.003). Subjects with the lower FT3 were more likely to die (Fig. 1).

### Cox proportional Hazard analysis for mortality

In univariate Cox regression analysis, age, sex, CI, COPD, Neoplasms, BMI, CC, and FT3 were related to mortality. All these significant factors were entered into the multivariate Cox regression analysis Model 2. Since FT3 was associated with CC and BMI, BMI and CC were excluded in model 1. In model 1, FT3 (HR, 0.65; 95% CI, 0.49–0.87, *P* = 0.004), Age (HR,1.06, 95% CI, 1.01–1.12, *P* = 0.011), Sex (HR, 0.41, 95% CI, 0.22–0.79, *P* = 0.008, male compared with female) and Neoplasms (HR, 2.41; 95% CI, 1.45–4.01, *P* = 0.001) were independent factors for the all-cause mortality of sarcopenia. In model 2, FT3 (HR, 0.63; 95% CI, 0.41–0.96, *P* = 0.031), sex (HR, 0.42, 95% CI, 0.19–0.95, *P* = 0.036, male compared with female) and CC (HR, 0.86; 95% CI, 0.76–0.97, *P* = 0.015) were independent factors for the all-cause mortality of sarcopenia (Table 3).

**Table 3:** Cox proportional hazard regression analysis of mortality

Variables	Univariate		Multivariate (Model 1)		Multivariate (Model 2)	
	HR (95%CI)	*P*	HR (95%CI)	*P*	HR (95%CI)	*P*
Age	1.07(1.03–1.13)	0.002	1.06(1.01–1.12)	0.011	1.03(0.98–1.09)	0.298
Sex	0.43(0.23–0.77)	0.005	0.41(0.22–0.79)	0.008	0.42(0.19–0.95)	0.036
Smoking	0.67(0.42–1.07)	0.190				
Drinking	1.31(0.41–4.15)	0.645				
CI	0.65(0.43–0.99)	0.043	1.16(0.72–1.87)	0.532	0.97(0.88–1.08)	0.557
COPD	1.75(1.14–2.68)	0.010	1.29(0.79–2.11)	0.294	1.39(0.78–2.45)	0.263
CHD	1.18(0.75–1.85)	0.479				
Diabetes	1.01(0.65–1.57)	0.969				
Hypertension	1.04(0.56–1.91)	0.907				
dyslipidemia	1.12(0.67–1.86)	0.672				
Neoplasms	0.59(0.37–0.96)	0.032	2.41(1.45–4.01)	0.001	1.77(0.94–3.32)	0.080
BMI	0.91(0.83–0.99)	0.025			0.97(0.88–1.07)	0.557
CC	0.81(0.76–0.87)	< 0.001			0.86(0.76–0.97)	0.015
HGS	0.97(0.94–1.01)	0.111				
FT3	0.43(0.31–0.59)	< 0.0001	0.65(0.49–0.87)	0.004	0.63(0.41–0.96)	0.031
FT4	0.95(0.88–1.03)	0.220				
TSH	1.01(1.00–1.01)	0.901				

Age, sex, CI, COPD, Neoplasms, FT3 were included in the multivariate model 1. Age, sex, CI, COPD, Neoplasms, BMI, CC, FT3 were included in the multivariate model 2

*Abbreviations*: *CI* Cerebral infarction, *COPD* Chronic obstructive pulmonary disease, *CHD* Coronary heart disease, *BMI* Body mass index, *CC* Calf circumference; HGS: Handgrip strength, *FT3* Free Triiodothyronine, *FT4* Free Thyroxine, *TSH* Thyroid stimulating hormone

## Discussion

In this study, we provided evidence that FT3 values were significantly lower in non-survival sarcopenic patients than in their survival counterparts. FT3 was positively associated with CC and HGS. Lower serum FT3 within the reference range was associated with all-cause mortality in older adults over 80 years, and the mortality increases with a decrease in FT3. To our knowledge, this is the first study to reveal the relationship between FT3 and all-cause mortality in patients with sarcopenia over the age of 80.

In our study, non-survivors had lower FT3 levels compared with survivors, while there was no significant difference between FT4 and TSH levels. Thyroid hormone seems to have high potential in predicting the outcome of clinical death, especially the active form of thyroid hormone FT3. Skeletal muscle has been identified as a direct target of thyroid hormone, which regulates stem cell proliferation and differentiation, contractile function, energy metabolism, and myofiber metabolism [18]. During myogenesis, the intracellular T3 concentration is precisely regulated by type 2 iodothyronine deiodinases (D2) and deiodinases type 3 (D3). Lower FT3 was found to be related to mortality in patients with chronic kidney failure [19], acute myocardial infarction [20], diabetic foot ulcers [21], and surgical sepsis [22]. Even recently, several studies showed that low FT3 serum levels were associated with the mortality of COVID-19 patients [23]. However, in most of these studies, the FT3 levels were not within the normal range, and patients with low FT3 may be a group of patients with serious diseases associated with high all-cause mortality. FT3 levels decrease with aging, suggesting that the thyroid may play a role in muscle aging [9]. Aging, decreased renal blood flow, malnutrition, metabolic disorder, and inflammatory cytokines increasing may lead to T3 levels decrease. In our study, all the participants had normal FT3 levels. However, we still found a relationship between FT3 levels and mortality in older adults with sarcopenia. TSH is considered the most sensitive indicator of thyroid dysfunction, and it regulates thyroid function through the hypothalamic-pituitary-thyroid axis but does not represent the biological activity of thyroid hormone. The biological activity of FT3 is much higher than that of FT4, which may be why FT4 and TSH were not found to be correlated with sarcopenia in this study.

Thyroid hormone abnormalities are common in critically ill patients. That is why we chose the participants whose thyroid hormones were within the reference range, avoiding low FT3 syndrome patients were included. Furthermore, to avoid the effect of illness on thyroid hormone levels, critical illnesses were excluded from this study, including severe infection, acute stroke, and acute myocardial infarction. However, many diseases may still affect thyroid hormone; even the thyroid hormone levels remain normal, which may be why the Kaplan Meijer curve showed a more significant decline in survival rate in the lowest tertile of the FT3 range in the first months.

Our study showed the serum FT3 level was positively related to CC and HGS, which are the index of sarcopenia. The muscle mass was measured by CC, which was supposed to be influenced by acute conditions, such as edema and bed-rest. Also, FT3 might be influenced by acute diseases. Thus, the patients with acute diseases and edema were excluded to reduce bias. The skeletal muscle is a vital target organ for thyroid hormones, which affect body composition and physical function via the FT3 receptor of mitochondria. Thyroid hormone increases mitochondrial protein synthesis and quantity, and increases mitochondrial basic oxygen consumption, ATP turnover, and maximum respiratory capacity [24]. A previous study showed that subjects with sarcopenia had a lower level of FT3. Furthermore, FT3 was positively related to skeletal muscle mass, muscle strength, and short physical performance battery scores. In contrast, neither FT4 nor TSH was associated with these parameters of sarcopenia in euthyroid subjects [11]. A study from Italy showed the highest quartile of FT3/FT4 ratio was associated with higher skeletal muscle index in a cohort of oldest-old (> 90 years), while the lowest quartile of FT3/FT4 ratio had worst physical performance compared to the higher groups [25].

Our results show that females with sarcopenia are at greater risk for mortality than males with the condition. However, the women percentage of the overall population who accepted healthcare at our division was lower than men, corresponding to a lower ratio of women included in this study, this imbalance may potentially affect the risk of mortality in sarcopenia. The gender aspect of sarcopenia has received little attention. A recent study showed that there were significant differences in fiber atrophy between men and women, and type II (“rapid contraction”) fiber atrophy only existed in men [26]. The mechanism of the gender difference is not entirely clear yet. Sex hormones play an essential role in maintaining skeletal muscle homeostasis, especially testosterone, a crucial synthetic factor in promoting muscle protein synthesis and regeneration [27]. This observation suggests that a more gender-specific analysis of sarcopenia is needed. Understanding the gender-related differences in sarcopenia is necessary to identify possible gender-dependent therapeutic targets.

This study has some strengths. First, individuals over 80 are usually excluded from many studies. However, this group had a higher sarcopenia prevalence. We are concerned about this group. Secondly, we completed the follow-up of all patients, and the longest follow-up time was 38 months. Therefore, this sample is representative and allows us to analyze problems in the population. Third, many comorbidities were adjusted when we analyzed the all-cause mortality risk.

This study also has some limitations. First, as an observational study, we cannot determine the causal relationship between serum FT3 levels and mortality. Secondly, this is a single-center study for hospitalized patients in the geriatric ward. The study’s participants are all Chinese, so the study’s results may not be applicable to other ethnic groups. Third, we did not assess the physical performance status, which indicates that severe sarcopenia may be more closely related to mortality. Finally, we only use CC to evaluate low muscle mass. Although EWGSOP2 mentioned that CC could be used when other methods cannot measure muscle volume, CC is not the gold standard of muscle volume.

## Conclusion

We found that FT3 was associated with calf circumference and handgrip strength. Moreover, FT3 was associated with mortality when controlling for chronic diseases, BMI and CC. As a result, FT3 is a prognostic determinant of sarcopenia. Routine evaluation of FT3 may be an easy method to identify high-risk patients with sarcopenia.

## Supplementary Information

**Additional file 1: Supplemental Fig. 1.** Flowchart for subjects enrolled in this study.

## Data Availability

Some or all datasets generated during and/or analyzed during the current study are not publicly available due to ethical concerns, but are available from the corresponding author on reasonable request.

## Abbreviations

FT3: Free Triiodothyronine
FT4: Free Thyroxine
TSH: Thyroid Stimulating Hormone
CC: Calf Circumference
HGS: Handgrip strength
COPD: Chronic Obstructive Pulmonary Disease
CHD: Coronary Heart Disease
BMI: Body Mass Index
EWGSOP: European Working Group on Sarcopenia in Older People
