# Supplementary material for: Lower serum FT3 within the reference range is associated with mortality for older adults over 80 years of age with sarcopenia
Source: BMC Geriatr. 2023 Feb 6;23:77. doi: 10.1186/s12877-023-03783-8 (PMC9900526; doi:10.1186/s12877-023-03783-8)
Supplement: Supplementary file 1 — Additional file 1: Supplemental Fig. 1. Flowchart for subjects enrolled in this study. [file 12877_2023_3783_MOESM1_ESM.docx]

**Supplemental Figure1: Flowchart for subjects enrolled in this study.**

Total participants aged≥80 years(n=1026)

Final samples (n=264)

Sarcopenia (n=475)

Exclusion

critical illness(n=97)

non-Sarcopenia(n=454)

Exclusion: (n=211)

thyroid hormones were not in the normal range(n=53)

diseases that may have effect on muscle metabolism (39)

thyroid disease (n=22)

presence of carcinomatous cachexia(n=37)

inability to communicate(n=13)

bedridden status(n=35)

edema(n=12)
